# Perceptions of education during COVID‐19 among agronomy, soil, and environmental science students

**DOI:** 10.1002/nse2.20055

**Published:** 2021-06-02

**Authors:** Colby J. Moorberg, Sarah Howe, Kevin J. Donnelly, Doohong Min

**Affiliations:** ^1^ Dep. of Agronomy Kansas State Univ. 2004 Throckmorton Hall, 1712 Claflin Rd. Manhattan KS 66502 USA

## Abstract

The COVID‐19 pandemic required changes in college course delivery, which may influence student perceptions of their education. We examined those perceptions among Students of Agronomy, Soil, and Environmental Sciences (SASES). The goals were to determine how changes in education during the COVID‐19 pandemic (a) changed student perceptions of online education, (b) shifted student comfort with online education and communication, and (c) highlighted issues limiting student success. A link to an online survey was sent to SASES members in late 2020 resulting in 31 responses. Course format shifted from mostly face‐to‐face delivery to synchronous and asynchronous online delivery following the COVID‐19 shutdown. Students perceived decreased instructor effort and increased student effort. Nearly all students had access to devices. However, access to fast, reliable internet was a common issue. A small percentage of students were more likely to take online courses after the pandemic, yet a majority were now more comfortable taking online classes and using virtual meeting software. Student concern for COVID‐19 was low. However, most reported wearing masks in buildings and on campus. Students reported increases and decreases in hours worked, with both changes resulting in perceived positive and negative academic impacts. These results should be considered by instructors and administrators planning instruction format changes during and after the COVID‐19 pandemic.

## Core Ideas

-  Students prefer face‐to‐face classes over online.
-  Students perceived a decrease in instructor effort and an increase in student effort.
-  Students became more comfortable taking online classes and meeting virtually.
-  Access to fast, reliable internet was a bigger issue than access to devices.

**Abbreviations**
SASES: Students of Agronomy, Soil, and Environmental Sciences

## 1 INTRODUCTION

The performance and satisfaction of students in courses delivered online vs. those delivered in face‐to‐face formats have been of interest in higher education since the advent of distance courses delivered online. However, the COVID‐19 pandemic with the sudden pivot of face‐to‐face courses to online delivery to facilitate social distancing created an unprecedented scenario for online education.

The effectiveness and student perceptions of online courses in natural sciences education were mixed prior to the COVID‐19 pandemic. Wuellner (2013) examined two undergraduate fisheries and wildlife sciences courses that were offered in both online and face‐to‐face formats and all taught by the same instructor. Wuellner found no difference in student satisfaction among the two offerings in one course, but in the other course, face‐to‐face students had worse student satisfaction. Further, Wuellner observed no difference in student performance with respect to grades, but did find differences with respect to Bloom's taxonomic categories. Online students performed better on knowledge assessments while face‐to‐face students performed better on comprehension and evaluation skills. Wuellner also found more instructor time per student was required for the online delivery compared to face‐to‐face delivery. Greenway and Makus (2014) examined student performance in an upper‐division agricultural economics course and found that online students performed marginally better than students taking the course face‐to‐face. This was attributed to the experience of the instructor teaching the course. Neu et al. (2017) examined learning gains and student satisfaction in two undergraduate animal science classes using pre‐ and post‐tests and student surveys. They found student satisfaction was high for both the face‐to‐face and online delivery modes, though student satisfaction was higher for face‐to‐face students in some instances. They also found student learning gains occurred in both delivery modes, though face‐to‐face students experienced greater learning gains in three of the four course years examined.

These examples show that online education is generally comparable to face‐to‐face delivery in natural science courses as related to student performance and student satisfaction. However, in each of these cases students had the ability to choose between online and face‐to‐face course delivery, and instructors likely had the ability to choose to teach through online delivery. During the COVID‐19 pandemic, the sudden pivot to online delivery forced many students and instructors to rapidly change their teaching and learning methods and strategies. Student perceptions of college course education during the COVID‐19 pandemic were of great interest.

Aristovnik et al. (2020) conducted a global study of 30,383 students from 62 countries. They found students in North America, Oceana, and Europe have exhibited the highest satisfaction with the pivot towards distance education methods during the COVID‐19 pandemic compared to other regions and continents. They also found students from North America, Oceana, and Europe perceived an increase in “workloads” (what we describe in this study as effort), and noted lack of computer skills and access to fast or reliable internet as significant hurdles towards their education following the shift to distance education.

Unger and Meiran (2020) surveyed undergraduate students at Wingate University (North Carolina) upon students’ return to online‐only delivery of an animal behavior course. They found many students (75.6%) had some level of anxiety towards the rapid shift to online education. They also found a majority of students felt preventative measures, such as the pivot to online, were based on good science. A follow‐up survey revealed anxiety toward online education had subsided somewhat following 3 weeks of distance education.

It is unclear if the student perceptions of higher education during the COVID‐19 pandemic described above apply to students in agronomy, crop, soil, and environmental sciences in the United States, or how student perceptions vary across multiple institutions. Thus, we set out to explore the perceptions of students who are members of Students in Agronomy, Crop, Soil, and Environmental Sciences (SASES).

## 2 METHODS

This study entailed a survey of members of SASES‐affiliated chapters from across the United States. Students in SASES are primarily enrolled in agronomy, crop science, soil science, and environmental science majors at bachelors‐granting institutions around the United States. The goals of this study were to determine how changes in education during the COVID‐19 pandemic have (a) changed student perceptions of online education, (b) shifted student comfort with online education and communication, and (c) highlighted issues that limit student success in remote education settings. The four investigators for this project are current faculty advisors to Wheat State Agronomy Club at Kansas State University, which is a SASES‐affiliated student organization.

The survey was conducted via Qualtrics. It was announced by staff of the American Society of Agronomy, Crop Science Society of America, and Soil Science Society of America (which operate SASES) in a newsletter to the SASES active member email list, and to the SASES advisor email list to be forwarded to the individual club/chapter email lists as a reminder for their students to respond. The survey was opened on 23 Nov.  2020 and closed on 21 Dec.  2020. Two reminders were sent to students and one to advisors during that time period.

The survey (available in Supplemental Material) was optimized for mobile screens, included 38 questions, and was predicted by the Qualtrics survey platform to take approximately 10 minutes or less to complete. The anonymous survey began with a required informed consent form and required demographic questions. Demographic questions included the name of college or university, name of major, year in school, gender, and whether students had taken an online class prior to the pandemic. The students were then asked a series of questions about the transition to distance education during the spring 2020 term for those who were enrolled in classes at the time. Topics of those questions included class format before and after the transition, satisfaction with the transition, effort from the instructor and students, access to devices and reliable internet connections, changes in employment, SASES club activity, and an overall reflective assessment of the spring term. A similar set of questions was asked for the fall 2020 term. Lastly, the respondents were asked to check a box for all statements that applied to them. These statements were split into two sets. The first focuses on general sentiments about the COVID‐19 pandemic. The second focuses on the impacts on the students' comfort with online education and digital technologies being used during the pandemic.

## 3 RESULTS

### 3.1 Demographics

**FIGURE 1:**
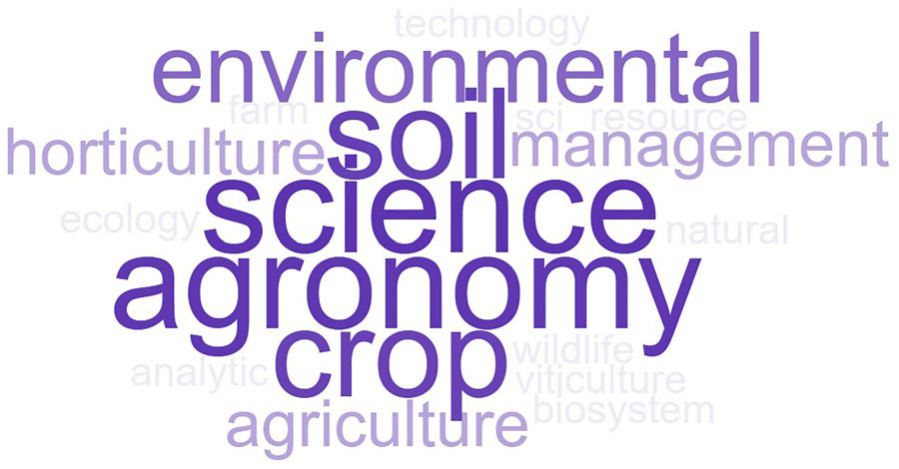
Word cloud of responses to the question, “What is the name of your major?” The more frequent a word occurred in the responses, the larger and darker that word is presented in the word cloud

There were 31 students that responded to this survey representing 21 different institutions of higher education (Supplemental Table S1), of which 19 were located in the United States, one in India, and one in South Africa. There were six respondents from Brigham Young University, Idaho and three from Kansas State University. All other institutions had one or two respondents. Students were asked to identify the name of their major. Responses are represented in a word cloud in Figure  1. Agronomy, Soil Science, Crop Science, and Environmental Science were the most common majors and disciplines represented, with many responses identifying more than one of those disciplines in the name of their major.

Of the 31 respondents, 18 (58%) were in their “4+” year of college, 8 (26%) were in their 3rd year, 3 (10%) were in their 2nd year, and 2 (6%) were in their 1st year. This distribution matches that of overall SASES membership (Supplemental Figure S1). Eighteen (58%) of the 31 respondents identified as female, 12 as male (39%), and 1 as non‐binary (3%). This also reflects the distribution of SASES membership, which includes 50% women, 48% men, and 2% individual identifying as non‐binary or that declined to identify a gender. Twenty of the 31 respondents (65%) noted they had taken at least one online class prior to the spring 2020 semester, with the remainder noting they had not previously taken an online class.

The 31 survey responses represented 6.4% of the total SASES membership at the time of this survey. Due to the small sample size, statistical tests were not performed to evaluate the impact of demographics on responses to survey questions.

### 3.2 Student perceptions of the spring 2020 pivot to online education

Five of the 31 survey respondents reported they were not enrolled in classes during the spring 2020 session. The survey logic thus skipped questions related to the spring 2020 session for those five students. The results of the 26 respondents who were enrolled in classes during the spring 2020 session are reported below. Two of those students did not answer all  questions.

**FIGURE 2:**
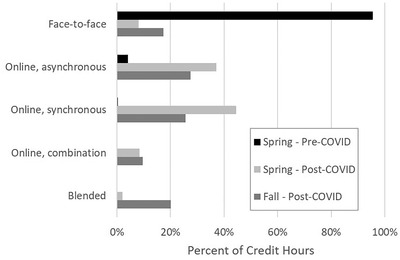
Distribution of course format as percent of credit hours for the pre‐ and post‐COVID spring session and the post‐COVID fall session. A total of 288 credits were reported for the pre‐ and post‐COVID spring session. A total of 324 credits were reported for the post‐COVID fall session

The format of courses taken by the students is shown in Figure 2. Students reported the number of credits in which they were enrolled for each of the five respective course formats. Reported credits were summed by format, and the percent was calculated for the spring pre‐COVID, spring post‐COVID, and fall post‐COVID sessions. A large majority (95%) of credits was face‐to‐face prior to the COVID‐19 pandemic, with some credits being taken in online asynchronous (4%) and online synchronous (<1%) formats. Following the arrival of the COVID‐19 pandemic during the spring 2020 session, courses shifted to predominately online formats (online asynchronous, 44%; online asynchronous, 37%). A smaller percentage of courses used a combination of asynchronous and synchronous online delivery (8%) and blended (2%) delivery. Some courses remained in face‐to‐face format (8%).

Students were generally unsatisfied with the transition to online education during the spring 2020 session. Out of 24 respondents, only 5 students reported being satisfied with the transition in all (1, 4%) or most (4, 17%) of their classes. Most (14, 58%) reported they were satisfied with the transition in some of their classes, and 5 (21%) reported not being satisfied with the transition in any of their classes.

There were mixed responses in the student perception of the effort being put into courses following the pivot to online education following the shutdown. Out of 24 respondents, 7 thought their instructors were putting in “much more” (4, 17%) or “more” (3, 13%) effort after the shutdown compared to 10 students who thought instructors were putting in “less” (8, 33%) or “much less” (2, 8%) effort after the shutdown. Seven students (29%) thought their instructors were putting in “about the same” amount of effort after the shutdown.

Student perceptions of their own efforts following the shutdown were also mixed. Out of the same 24 respondents, 8 thought they were putting in “much more” (4, 17%) or “more” (4, 17%) effort after the shutdown compared to 8 that thought they were putting in “less” (5, 21%) or “much less” (3, 13%) effort after the shutdown. Eight (33%) thought they were putting in “about the same” amount of effort after the shutdown.

Students generally had access to devices and internet to access their coursework. Only 1 student out of 24 reported not having a non‐cell phone device to access their schoolwork. Two students (9%) had to share their non‐cell phone device with a sibling, other family member, or roommate. Students reported that they primarily accessed their school work from home (11, 46%) or their college dorm room or apartment (13, 54%). No students reported accessing school work from other locations such as coffee shops or other public Wi‐Fi hotspots. Seventy‐nine percent of students reported that the speed or reliability of their internet connection regularly (7, 29%) or occasionally (12, 50%) interfered with their ability to participate in classes or complete homework. Only five students (21%) reported no interruptions with their internet connection.

**FIGURE 3:**
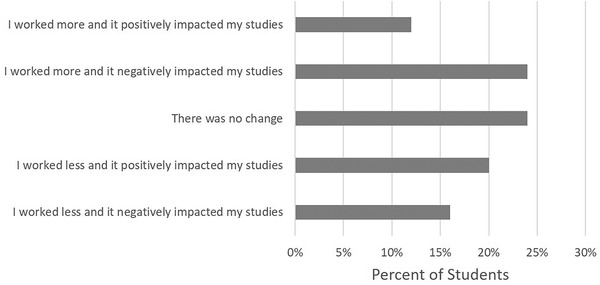
Impact of changes to hours worked at a job on the students’ studies. Results are reported as a percent of total respondents, *n* = 24

The number of hours students worked before and after the shutdown was similar. The median hours worked before the shutdown was 12.5 hours compared to 15 hours after the shutdown. The impact from changes in hours worked had very mixed impacts on the students’ schoolwork, as shown in Figure 3.

There were mixed results related to the continued operation of SASES chapters as well. Ten students (45%) reported that their SASES chapter continued to meet virtually after the shutdown in the spring session, while 12 (55%) reported that their chapters ceased to meet.

Students were asked to select the option that best completes the sentence: "Overall the transition to online education in the spring of 2020 ____________.” None of the students reported that the transition to online went well, while 13 (54%) reported it “went okay”, 8 (33%) reported it “went poorly”, and 3 (13%) reported it “went terribly”. The students were asked to explain their answer to that question in a comment box. All responses are listed in Table 1.

**TABLE 1:** Comments explaining responses to the summary question on how the spring transition to online education went. Immediately preceding this question, students were asked to select the option that best completes the sentence: “Overall the transition to online education in the spring of 2020 ____________.” With the options including “went well”, “went okay”, “went poorly”, and “went terribly”. All responses are listed in their entirety

Comments
“The instructors expected us to continue the same course work load even though the lectures were confusing. One of my professors never answered any questions during class and it made the experience awful.”
“I did not receive the education I paid for.”
“Definitely some bumps, but the teachers did the best they could.”
“I don't think I was prepared for online learning, it was hard to focus and do everything virtually all of a sudden.”
“Some professors did an excellent job in the 1 week they had to prepare for online learning for the rest of the semester, while other professors didn't seem to care about putting in the effort to make online learning work. The success of an online class lies mainly on how much effort the professor is willing to put into it.”
“Many professors did not transition very well or didn't know how to put the information we were learning online because it was hands on.”
“I was struggling with teaching myself how to do my course work because I didn't want to go on a zoom call with my professors to ask them questions. I don't like that online meeting, I would like to meet with them in person and get help that way. My parents also encouraged me to work since a lot of business were hiring due to the rise in bull shopping so balancing working 40 hours a week and my homework was difficult. Overall I hate online classes and would rather have them in person.”
“The transition to online education made me lose motivation, but I still completed my assignments and finished the semester with a decent grade point average (after pass and fail was enacted).”
“I would have preferred everything to be asynchronous– I think it would be easier that way.”
“I just am not very good with online classes so my grades dropped significantly when everything went online.”
“I have taken online classes before, I knew what to expect from them.”
“Because end of it all I managed to pass though i didn't get distinctions ”
"It is very difficult to raise one's hand and ask a question on any type of online/virtual class, so the questions I had during class often went unanswered. The level of attention that I had for classes drastically decreased. Overall I feel like my learning decreased.”
“I simply don't care for online classes.”
“The amount of work I was required to do was insane. It felt like the teachers gave up completely and left it to us to learn and get the material. I was unable to access help from them often. It was overwhelming, and I failed a couple classes. I've questioned my drive to graduate many times now because I wish I was learning like I used to before the pandemic.”

### 3.3 Student perceptions of the fall 2020 semester

There were 26 respondents who reported they were enrolled in classes for the fall academic session and answered questions related to that term.

The format of classes during the fall session are shown in Figure 2. The most common formats were “online, synchronous” (44%) and “online, asynchronous” (37%), followed by “blended” (20%), “face‐to‐face” (17%), and “online, combination of asynchronous and synchronous”  (10%).

All students reported having access to a non‐phone device to complete homework and participate in classes during the fall session. Two out of 24 students (8%) reported they had to share their non‐phone device with a sibling, other family member, or roommate during the fall academic session. All students reported the primary location where they accessed online coursework and materials was from their place of residence during the fall semester, and none reported primarily accessing their online coursework and materials from on‐campus (e.g., library) or off‐campus (e.g., coffee shop) public Wi‐Fi hotspots. Sixty‐six percent of students reported that their internet connection regularly (6, 25%) or occasionally (10, 42%) interfered with their ability to participate in classes or complete homework. Eight students (33%) reported no interruptions with their internet connection.

Students worked a comparable number of hours per week (10 hours per week, median) to the number of hours worked during the spring session before and after the shutdown.

**FIGURE 4:**
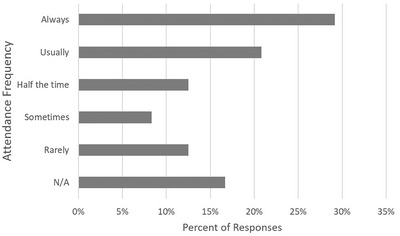
Self‐reported in‐person attendance for classes that offered remote participation as an alternative to in‐person attendance. There were 24 responses to this question. The N/A category represents students who identified “I do not have virtual attendance as an alternative to in‐person attendance”

Many students noted a preference for in‐person classes in their comments in Tables 1 and  2. For the fall session students were asked, “For classes that allow virtual attendance as an alternative to in‐person attendance, how often do you attend in person?” Half of students reported they “always” (7, 29%) or “usually” (5, 21%) attended in‐person for classes where online participation was provided as an alternative to in‐person attendance (Figure 4). Others reported attending in‐person half the time (3, 13%), sometimes (2, 8%), or rarely (3, 13%), with 17% (4) reporting that this question did not apply as they did not have virtual attendance as an alternative to in‐person attendance.

**TABLE 2:** Comments explaining responses to the summary question on how the fall session is going. Immediately preceding this question, students were asked to select the option that best completes the sentence: “Overall the fall 2020 term is ________.” With the options including “going well”, “going okay”, “going poorly”, and “going terribly”. All responses are listed in their entirety

Comments
“The professors have learned more about teaching virtually and are more understanding with internet problems.”
“I am not getting the education I pay for. The blended options are horrible. Classes need to be one way or the other, either all online or all face to face. Also, the more asynchronous a class the better. It is especially hard to do online classes synchronously.”
“I've done online classes before, so it isn't a new challenge, but classes that were made online quickly are much harder than usual because we're given more work to compensate for our lack of being in person. Usually that extra work is both taxing and unnecessary.”
“I expected it this time, so it's not that bad.”
“I'm more adjusted to online learning but it still isn't working for me.”
“I managed all A's, so that means it has gone well for me. Professors overloaded classes at the beginning semester, but backed off towards the end.”
“Professors still don't know how to use technology. There are a lot of technology issues on campus, in the classroom, at the professor's home, and my own home. I am not getting the same experience in my classes as I would if they were all on campus.”
“Online class is not a good option for me but I have been able to get my work done. However, the overall experience is very stressful.”
“It is going better than spring 2020 but thankfully my Ag classes were in person which I found better than learning virtually. My gen ed courses were all online and it has been difficult to do those cuz i do not do well with online learning. Thankfully, we were still able to live in our residence halls so my friends were enrolled in the same classes I was so it was helpful doing homework with them.”
“One teacher emailed us saying she had no motivation to teach this semester. She has only posted 5 hours' worth of material not even lectures, just websites for us to look at and one class still has yet to post anything.”
“Similar to spring 2020, I am having a hard time finding motivation to do well in school.”
“I am way too depressed and anxious and grieving to be doing well in classes this semester– especially when professors are still strict with deadlines and absences as if nothing is different.”
“Online school makes it hard to focus. Mental health was an issue.”
“I would much prefer to have all my classes in person but I guess we just have to do what local government is telling us to do.”
“It was better than I expected.”
“I typically enjoy school a lot. Because I enjoy I learn a lot and it feels fulfilling. I still have good grades this semester, but I feel like I have learned comparatively much less than previous semesters and it has been much less fulfilling. I feel like the instructors don't often know how to keep class engaging while meeting virtually; some of them seem to be struggling as much as the students. I am not saying it is the teachers' fault, I feel like most are doing their best. It also has to do with staying focused and engaged in the lesson. It's really easy to not pay attention when class is a Zoom meeting.”
“Most of my classes are strictly online, either meeting over zoom, or just online with no zoom, and for me it has been so hard. My grades have never been this bad.”
“Still don't really feel that I learn from online classes.”
“I'm doing SO WELL in my classes that are offered in person. I feel motivated and I feel like I understand the material well. But my classes online, I am struggling to pass. I don't think I will fail a class like I did last semester, but I am so close to failing a couple online classes. The stress is so overwhelming when it comes to online learning.”

Seventy‐seven percent of students (17) reported their SASES chapter was meeting during the fall session, including face‐to‐face (3, 14%), virtually (3, 14%), mix of face‐to‐face and virtually (9, 41%), and simultaneously face‐to‐face and virtually (2, 9%). Five students (23%) reported their SASES chapter was not meeting during the fall session.

Students were asked to complete the sentence, “Overall the fall 2020 term is ________.” A majority of students reported that the fall semester was “going okay” (16, 66%) or “going well” (2, 8%) with the remainder of students reporting the fall semester was “going badly” (3, 13%) or “going terribly” (3, 13%). All responses are listed in Table 2.

### 3.4 General student perceptions of COVID‐19

**FIGURE 5:**
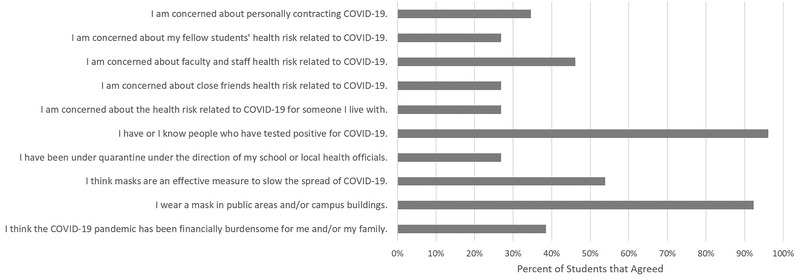
Percent of students that agreed with statements about the COVID‐19 pandemic. Students were asked to check a box next to each of the ten statements that applied. Twenty‐six students responded to this question

**FIGURE 6:**
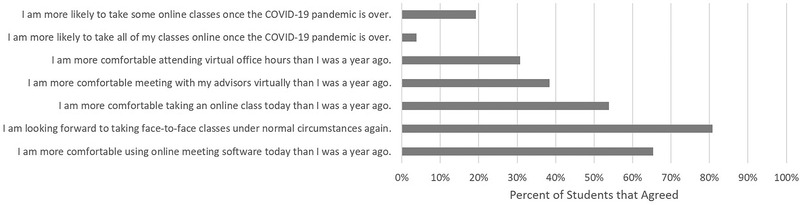
Percent of students that agreed with statements about outcomes from the COVID‐19 pandemic. Students were asked to check a box next to each of the seven statements that applied. Twenty‐six students responded to this question

The students were asked to check boxes next to a series of questions that applied, as shown in Figures 5 and 6. Less than half of the students were concerned about contracting COVID‐19 (9, 34%); health risks that COVID‐19 posed to their fellow students (7, 27%), faculty and staff (12, 46%), close friends (7, 27%), or someone they live with (7, 27%). Ninety‐six percent (25) had or knew someone who had tested positive for COVID‐19. Twenty‐seven percent (7) of students reported having to go into quarantine at the direction of school or local health officials. A slight majority (14, 54%) thought masks were an effective measure to slow the spread of COVID‐19, though 92% (24) reported they wore masks in public or campus buildings. Thirty‐eight percent (10) of students thought the COVID‐19 pandemic had been financially burdensome on their families.

As shown in Figure 6, only a small minority of students (5, 19%) reported they would be more likely to take some or all of their classes online once the pandemic is over. However, 54% of students (14) reported they were more comfortable taking online classes than a year ago, and 65% (17) reported being more comfortable using virtual meeting software than a year ago. Less than 50% reported they were more comfortable attending virtual office hours (8, 31%) or meeting virtually with their advisor (10, 38%) than a year ago. Eighty‐one percent of students (21) reported looking forward to returning to face‐to‐face instruction once the pandemic is over.

## 4 DISCUSSION

The sample size of 31 respondents to this survey is small, which was likely the result of the timing of this survey coinciding with the end of the fall 2020 academic session for most institutions. However, the pool of respondents is representative of overall SASES membership demographics for gender and year in school based on data from SASES (Supplemental Figure S1). Twenty‐nine of the 31 respondents were from U.S. institutions of higher education. Thus, this survey should provide useful insight to student perspectives of higher education during the COVID‐19 pandemic, especially for natural science majors and majors associated with SASES in the United  States.

The course formats used during the fall session were more diverse than class formats offered in the spring both before and after the pivot to online instruction. This is likely the result of instructors having more time to prepare courses for online delivery, as well as having the option for face‐to‐face and hybrid delivery of their classes. The most common format during the COVID‐19 pandemic was synchronous delivery of online courses. This corresponds to findings by Aristovnik et al. (2020) who found 59.4% of students worldwide participated in classes predominately through what the authors termed “real‐time video conferences”, which is analogous to “synchronous online education” used in this survey.

The very abrupt transition of face‐to‐face courses to online delivery during the spring session likely played a role in the general dissatisfaction and anxiety among students about the pivot to online instruction. Among the causes of stress and points of contention listed in Table 1 were issues with internet access, student and instructor familiarity with technology, difficulty in asking questions or meeting with instructors, lack of motivation among students and instructors, and general preference for face‐to‐face instruction. These issues were also highlighted in the results from the global survey by Aristovnik et al. (2020). Anxiety about the pivot to online education was also noted by Unger and Meiran (2020) in their survey of animal science students at Wingate University. The survey used by Unger and Meiran occurred at multiple times during the spring semester, and they showed that anxiety subsided somewhat following 3 weeks of online education. Results of our survey conducted at the end of the fall 2020 academic session suggest the anxiety never went away completely, and that it even persisted into the fall 2020 session.

Students’ comments regarding the fall session focused primarily on their preference for face‐to‐face class format, a general lack of motivation, and concerns for their own mental health. These issues should be a focus of instructors and administrators as the pandemic continues, as these issues represent a significant hurdle to student success during the COVID‐19 pandemic.

There were more students who thought their instructors’ amount of effort decreased following the pivot to online than students who thought their instructors’ efforts increased. This perception is likely not aligned with reality. Wuellner (2013) found that online classes required more time per student than face‐to‐face delivery. Van de Vord and Pogue (2012) found that face‐to‐face teaching required more time per student. However, they concluded that certain aspects of online teaching take considerably more time per student than face‐to‐face delivery. However, that study examined existing online courses and did not measure time required to create content for online courses. During and following the shift to online education as a result of the pandemic, many instructors were having to create online content for courses traditionally taught face‐to‐face. Time required for such activities is significant. These activities are not directly observed by students, and thus do not influence student perceptions of instructor effort.

Student's perceptions of the amount of work they were putting into their coursework was mixed. The mixed perceptions of student effort likely reflect variability in the ways that instructors transitioned their classes to distance education following the shutdown, overall student motivation, and changes in working hours at part‐time jobs. In contrast, Aristovnik et al. (2020) found 54.7% of students from North America perceived an increase in course workload.

Loss of income can be a significant stressor to college students, especially if they depend on that work for money for groceries, rent, tuition and fees, and other college expenses. Students who work while attending college can face additional anxiety compared to non‐working students (Mounsey et al., 2013). Changes in employment status or hours worked can add an additional level of anxiety and stress. The results presented here showed that shifts in working hours as a result of the COVID‐19 pandemic impacted students differently, both positively and negatively. Part‐time work and finances should be a consideration for instructors and administrators when helping underperforming students during and after the pandemic.

Access to reliable and fast internet proved to be more of a concern than access to devices. Only a small number of students reported not having access to a non‐mobile phone device or having to share those devices with others. In contrast, a majority of students reported that the speed and reliability of their internet connection was a hindrance to participating in the classes or coursework in both the spring and fall semesters. The number of students reporting issues with their internet during the fall semester did decrease relative to the spring. This is likely the result of more students living on or near campus with better access to reliable and fast internet connections. Many students of agronomy, soils, and environmental science come from rural areas (McCallister et al., 2005; Miller, 2011) where access to reliable and fast internet connections is often a concern (Lai & Widmar, 2020). Aristovnik et al. (2020) and Mardis (2013) also reported that students from rural areas globally had problems with access to reliable internet during the COVID‐19 pandemic.

During the fall session many institutions allowed classes to be administered in face‐to‐face formats. In many cases, classes were offered in hybrid formats where students could or had to participate partially online to compensate for limited classroom capacities, quarantines, sickness, or protection for at‐risk students and faculty. A notable portion of students attended only half the time or less when given the opportunity to attend in‐person. Such attendance is in contrast with the noted preference for face‐to‐face delivery of courses that showed throughout this survey. This could be due to scheduling difficulty during the fall session associated with some classes only being offered online, and thus requiring participation by the student from their residence. If students have back‐to‐back classes where one is online only, and one is offered in a hybrid format, it is easier to attend both without being late if the student can avoid having to rush from their residence to campus in a short amount of time. Poor attendance at in‐person classes could also be associated with students actively minimizing their risk of exposure to COVID‐19, minimizing the risk of transmission to others, or reluctance to wearing masks inside of campus buildings.

Many SASES‐affiliated chapters were not active following the shutdown during the spring semester. More chapters met during the fall semester, often in hybrid or virtual meeting formats. Notably, few met face‐to‐face only. The authors participated in the SASES annual meeting that was held virtually in November, and we noted that participation in the virtual meeting was very low compared to in‐person meetings we had attended in years prior. Causes of this low participation in the national SASES meeting, and perhaps at local chapter meetings should be explored in more detail to improve virtual participation as this pandemic continues, and for encouraging virtual participation once the pandemic concludes. Student engagement in extracurricular activities during this pandemic may play a role in student satisfaction and overall mental well‐being. Unfortunately, the small sample size from this survey prevents us from determining if continued meeting of SASES chapters influenced student perceptions of higher education during the spring and fall semesters of 2020.

Few students expressed concern about COVID‐19. This includes personally contracting the virus, or the health risks of fellow students, faculty and staff, close friends, or someone the student lives with. This likely reflects the low risk COVID‐19 poses to college‐age individuals. Most students did report they knew someone who had tested positive for the virus. The risk perceived by these students could be influenced by knowing fellow college‐aged individuals that recovered from the virus quickly and not knowing individuals who experienced more difficult recoveries or did not recover.

A slight majority of respondents thought masks are an effective measure to slow the spread of COVID‐19. The proportion of students agreeing to that statement is low, considering masks have been shown to be an effective strategy in slowing the virus (Centers for Disease Control & Prevention, 2020; Van Dyke et al., 2020). Colleges and universities should increase their efforts to educate students about the effectiveness of masks. A high percentage of students did report wearing masks in campus buildings. This is likely the result of compliance with campus mask mandates. Aristovnik et al. (2020) also found a high percentage (86.7%) of students reported wearing masks, along with other changes in hygiene and habits aimed at reducing the spread of the virus.

Exposure to online education as a result of the pandemic did not have a great effect on student perceptions of online courses. Only a small percentage of students reported they would be more likely to take online classes once the pandemic is over. However, a majority of students reported they feel more comfortable about taking classes online now compared to a year ago. Similarly, a majority of students reported they are more comfortable using virtual meeting software now compared to a year ago. Students’ preference for face‐to‐face class formats and their increased comfort with virtual meeting applications should both be weighed in decisions related to permanent changes in class formats after the pandemic is over. Similarly, only a minority of students reported being more comfortable with virtual office hours or virtual meetings with advisors, so implementing changes in format to those crucial parts of the undergraduate experience should be approached with caution.

## 5 CONCLUSIONS

The changes required to continue delivery of college education during the COVID‐19 pandemic have the potential to greatly influence student perceptions of their college education, views towards modes of education, and behavior to limit the spread of the virus. This study examined those changes among students in agronomy, crop, soil, and environmental sciences primarily from U.S. bachelors‐granting institutions. Prior to the COVID‐19 pandemic students were primarily enrolled in face‐to‐face classes. Those courses shifted to synchronous and asynchronous online delivery as a result of the shutdowns associated with the COVID‐19 pandemic. The fall 2020 session and the return of students to college campuses across the United States facilitated additional modes of delivery in the fall. Students voiced an overall preference for face‐to‐face courses. During the spring 2020 session when most classes shifted to online delivery, students perceived a decrease in effort by their instructors and perceived an increase in their own effort. Nearly all students had access to non‐mobile phone devices to participate in classes remotely. However, a majority of students reported that access to fast and reliable internet connections impacted that participation. Only a small percentage of students reported they would be more likely to take online courses after the pandemic is over. However, a majority of students reported being more comfortable taking online classes and using virtual meeting software than they were a year prior. Only a small number of students reported being more comfortable with attending virtual office hours or virtual meetings with advisors. Instructors, advisors, and institutions should therefore use caution when imposing shifts to virtual formats for these important interactions with students. A high proportion of students reported wearing masks in buildings and on campus despite a slim majority (54%) reporting agreement that masks are an effective measure to slow the spread of the virus and a low overall concern for COVID‐19.

The pandemic has also impacted student extracurricular activities. Many students reported changes in hours worked following the shutdown during the spring 2020 session, but those changes impacted each student differently. Some students experienced increases in weekly hours worked resulting in negative impacts on their academics, while about an equal number reported a positive impact. The same trend was evident for those that worked less. Most SASES chapters were not actively meeting following the shutdown in the spring 2020 session, and few chapters were meeting face‐to‐face during the fall 2020 session. This decreased chapter activity may relate to the low level of participation in the national SASAS virtual meeting in November 2020. Thus, SASES chapters should focus on increasing participation in upcoming academic sessions.

## AUTHOR CONTRIBUTIONS

Colby J. Moorberg: Conceptualization; Formal analysis; Investigation; Methodology; Project administration; Visualization; Writing‐original draft; Writing‐review & editing. Sarah Howe: Conceptualization; Formal analysis; Methodology; Writing‐review & editing. Kevin J. Donnelly: Conceptualization; Formal analysis; Methodology; Writing‐review & editing. Doohong Min: Conceptualization; Formal analysis; Methodology; Writing‐review & editing

## CONFLICT OF INTEREST

The authors declare no conflict of interest.

## Supporting information

Supporting Information
