# Supplementary material for: Perceptions of education during COVID‐19 among agronomy, soil, and environmental science students
Source: Natural Sciences Education. 2021 Jun 2;50(1):e20055. doi: 10.1002/nse2.20055 (PMC8206661; doi:10.1002/nse2.20055)
Supplement: Supplementary file 1 — Supporting Information [file NSE2-50-e20055-s001.pdf]

Perceptions of education during COVID-19 among agronomy, soil, and environmental science students

Colby J. Moorberg, Sarah Howe, Kevin J. Donnelly, Doohong Min.

Supplemental Information

SI Table 1. Number of Responses by Institution.

Name of College or University	Responses
Banaras Hindu University	1
Brigham Young University	1
Brigham Young University, Idaho	6
California State University, Fresno	1
Dickinson State University	1
Iowa State University	2
Kansas State University	3
Michigan State University	1
Mississippi State University	1
New Mexico State University	1
Purdue University	1
South Dakota State University	1
Tennessee State University	1
Universidad de Puerto Rico Recinto de Mayagüez	1
University of Arizona	2
University of Forthare	1
University of Wisconsin, Madison	1
University of Wisconsin, Platteville	2
University of Wisconsin, River Falls	2
Western Illinois University	1

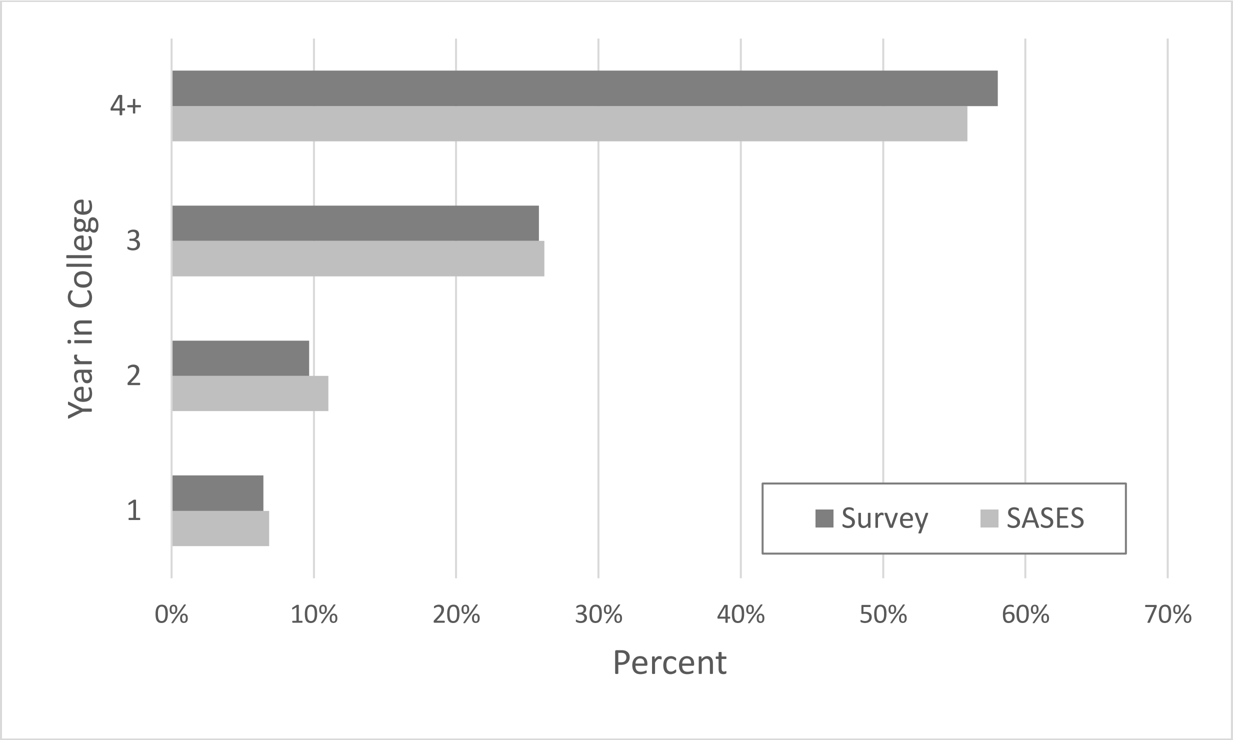

SI Figure 1. Distribution of survey respondents and SASES members by year in college. There were 31 survey respondents and 481 members of SASES at the time of the survey. The year in school for SASES membership was estimated based on student reported projected graduation date. SASES member data courtesy of Beth Jacques and the American Society of Agronomy, Crop Science Society of America, and Soil Science Society of America.

Qualtrics Survey

Consent

Introductory Information and Consent:
Welcome to the Students of Agronomy, Soils, and Environmental Science (SASES) COVID-19 Survey. This survey is designed to collect information about the views of undergraduate students who were enrolled in college classes during the spring and/or fall terms of 2020 and who are members of SASES chapters. The goals of this survey are to determine how changes in education during the COVID-19 pandemic has i) changed student perceptions of online education, ii) shifted student comfort with online education and communication, and iii) influenced student success in remote education.

The results from this survey may be published as an open-access journal article in a peer-reviewed teaching journal. Results from this survey will be used by instructors to inform decisions on class delivery format and modes of interaction with students during and after the COVID-19 pandemic.

This project is research, and your participation in this survey is voluntary. You may withdraw your consent at any time and stop participating at any time without explanation, penalty, or loss of benefits or academic standing.

Approximate Time to Complete: 10 minutes

Contact Information:
Survey intent and outcomes: Colby J. Moorberg, PhD, CPSS (principle investigator, moorberg@ksu.edu, 785-532-7207), Sarah Howe, MS (co-investigator, showe@ksu.edu, 785-532-7258), Kevin Donnelly, PhD (co-investigator, kjd@ksu.edu, 785-532-5402), and Doohong Min, PhD (co-investigator,

dmin@ksu.edu,785-532-2292). Qualtrics functionality and content: K-State IT Helpdesk (helpdesk@k-state.edu, 785-532-7722). If you have questions about your rights or welfare as a participant, please contact Rick Scheidt (Chair, Committee on Research Involving Human Subjects, rscheidt@ksu.edu, 785-532-3224) or Cheryl Doerr (Associate Vice President for Research Compliance, cdoerr@ksu.edu, 785-532-3224). Based upon information provided to the Kansas State University Institutional Review Board, this activity was classified as exempt on _____________, _____, 2020 under the criteria set forth in the Federal Policy for the Protection of Human Subjects, 45 CFR 46.101(b)(1).

By selecting “I agree” below, you verify that you read and understand the information provided above, and willingly agree to participate in this study under the terms described, and acknowledge that you may print a copy of this consent form to retain for your own records, and that you are at least 18 years of age.

- ○ I agree
- ○ I do not agree

Demographics

What is the name of your college or university?

____

What is the name of your major?

____

What is your year in college?

- ○ Year 1
- ○ Year 2
- ○ Year 3
- ○ Year 4+

What is your gender?

- ○ Male
- ○ Female
- ○ Non-binary

Have you ever taken an online class before Spring 2020?

- ○ Yes
- ○ No

Terms

This survey will use the following terms and definitions:

- **Face-to-face** - course delivery in which course content is delivered primarily with the class and instructor attending in person with accommodations to facilitate remote participation by students or the instructor if needed due to COVID-19 interruption
- **Online, synchronous** - course delivery in which the instructor and students meet regularly online in real-time meetings for lecture or other activities
- **Online, asynchronous** - course delivery in which tudents are rarely, if ever, required to meet in a synchronous setting and instead complete asynchronous learning activities and engagement on their own time
- **Online, synchronous/asynchronous combination** - course delivery in which

part of the instruction each week is delivered using synchronous delivery and the remainder is delivered asynchronously
- **Blended** - course delivery in which a mix of face-to-face and online delivery is used
- **SASES** - Students of Agronomy, Soils, and Environmental Science

Spring 2020 Questions

Were you enrolled in college classes during the spring 2020 term?

○ Yes
○ No

Please answer the questions below based on the spring 2020 term.

Please indicate the number of credits from classes that predominately used the following delivery modes BEFORE the COVID-19 shutdown.

Face-to-face	0
Online, asynchronous	0
Online, synchronous	0
Online, synchronous/asynchronous combination	0
Blended	0
Total	0

Please indicate the number of credits from classes that predominantly used the following delivery modes AFTER the COVID-19 shutdown.

Face-to-face	0
Online, asynchronous	0
Online, syncronous	0
Online, synchronous/asynchronous combination	0
Blended	0
Total	0

For how many of your classes were you satisfied with the transition to online following the COVID-19 shutdown?

- ○ All classes
- ○ Most classes
- ○ Some classes
- ○ None

From your perspective, how much time and effort did your instructors put into their class after the shutdown compared to before the shutdown?

- ○ Much more after the shutdown
- ○ More after the shutdown
- ○ About the same
- ○ Less after the shutdown
- ○ Much less after the shotdown

From your perspective, how much time and effort did you put into your coursework after the shutdown compared to before the shutdown?

- Much more after the shutdown
- More after the shutdown
- About the same
- Less after the shutdown
- Much less after the shotdown

After the shutdown did you have access to a non-phone device to complete your coursework and participate in or watch lectures?

- Yes
- No

Did the device you used need to be shared with anyone else, such as a sibling, other family member, or a roommate?

- Yes
- No
- I did not have access to a non-phone device

Where did you primarily access your coursework and materials following the transition to online delivery?

- Home
- College dorm or apartment
- Other (e.g. coffee shop or other public WiFi)

Did the speed or reliability of your internet service interfere with your ability to participate in classes or complete homework?

- Yes, regularly
- Yes, occasionally
- No

How many hours did you work per week BEFORE the shutdown?

- 0 hours
- 5 hours
- 10 hours
- 15 hours
- 20 hours
- 25 hours
- 30 hours
- 35 hours
- 40 hours
- Over 40 hours

How many hours did you work per week AFTER the shutdown?

- 0 hours
- 5 hours
- 10 hours
- 15 hours
- 20 hours
- 25 hours
- 30 hours
- 35 hours
- 40 hours
- Over 40 hours

Reflecting on the semester, how did any change in work hours impact your studies?

- ◯ I worked more and it positively impacted my studies
- ◯ I worked more and it negatively impacted my studies
- ◯ There was no change
- ◯ I worked less and it positively impacted my studies
- ◯ I worked less and it negatively impacted my studies

Did your SASES-affiliated club or chapter continue to meet virtually after classes pivoted online?

- ◯ Yes
- ◯ No

Select the option that best completes the following sentence: "Overall the transition to online education in the spring of 2020 ____________.

- ◯ Went well
- ◯ Went okay
- ◯ Went poorly
- ◯ Went terribly

Please explain your answer to the previous question.

Fall 2020 Questions

Are you enrolled in classes during the fall 2020 term?

- ○ Yes
- ○ No

Please answer the questions below with respect to the fall 2020 term.

Please indicate the number of credits from classes that use the following delivery modes during the fall 2020 term.

Face-to-face	0
Online, asynchronous	0
Online, synchronous	0
Online, synchronous/asynchronous	0
Blended	0
Total	0

How many of your classes have changed the delivery mode since the start of the fall term?

- ○ All classes
- ○ Most classes
- ○ Some classes
- ○ Non

Do you have access to a non-phone device to complete your coursework and participate in or watch lectures?

- ◯ Yes
- ◯ No

Does the non-phone device you use need to be shared with anyone else, such as a sibling, other family member, or a roommate?

- ◯ Yes
- ◯ No
- ◯ I do not have access to a non-phone device

Where do you primarily access your coursework and materials?

- ◯ From where I live during the school term
- ◯ Library or other campus locations
- ◯ Other (e.g. coffee shop or other public WiFi)

Does the speed or reliability of your internet service interfere with your ability to participate in classes or complete homework?

- ◯ Yes, regularly
- ◯ Yes, occasionally
- ◯ No

How many hours are you working per week during the fall 2020 term?

- 0 hours
- 5 hours
- 10 hours
- 15 hours
- 20 hours
- 25 hours
- 30 hours
- 35 hours
- 40 hours
- Over 40 hours

For classes that allow virtual attendance as an alternative to in-person attendance, how often do you attend in person?

- Always
- Usually
- Half the time
- Sometimes
- Rarely
- I do not have virtual attendance as an alternative to in-person attendance

Is your SASES-affiliated club or chapter continuing to meet during the fall term?

- Yes, face-to-face
- Yes, virtually
- Yes, mix of face-to-face and virtually
- Yes, simultaneous face-to-face and virtually
- No

Select the option that best completes the following sentence: "Overall the fall 2020 term is ________"

- ○ Going well
- ○ Going okay
- ○ Going poorly
- ○ Going terribly

Please explain your answer to the previous question.

COVID-19 Statements

Check all of the following statements that apply.

- ☐ I am concerned about personally contracting COVID-19.
- ☐ I am concerned about my fellow students' health risk related to COVID-19.
- ☐ I am concerned about faculty and staff health risk related to COVID-19.
- ☐ I am concerned about close friends health risk related to COVID-19.
- ☐ I am concerned about the health risk related to COVID-19 for someone I live with.
- ☐ I have or I know people who have tested positive for COVID-19.
- ☐ I have been under quarantine under the direction of my school or local health officials.
- ☐ I think masks are an effective measure to slow the spread of COVID-19.
- ☐ I wear a mask in public areas and/or campus buildings.
- ☐ I think the COVID-19 pandemic has been financially burdensome for me and/or my family.

COVID-19 Statements Continued

Check all of the following statements that apply.

- ☐ I am more likely to take some online classes once the COVID-19 pandemic is over.
- ☐ I am more likely to take all of my classes online once the COVID-19 pandemic is over.
- ☐ I am more comfortable attending virtual office hours than I was a year ago.
- ☐ I am more comfortable meeting with my advisors virtually than I was a year ago.
- ☐ I am more comfortable taking an online class today than I was a year ago.
- ☐ I am looking forward to taking face-to-face classes under normal circumstances again.
- ☐ I am more comfortable using online meeting software today than I was a year ago.

Powered by Qualtrics
